# Characterization of Novel Exopolysaccharides from *Enterococcus hirae* WEHI01 and Its Immunomodulatory Activity

**DOI:** 10.3390/foods11213538

**Published:** 2022-11-07

**Authors:** Kaiying Jia, Min Wei, Yao He, Yujie Wang, Hua Wei, Xueying Tao

**Affiliations:** State Key Laboratory of Food Science and Technology, Nanchang University, Nanchang 330047, China

**Keywords:** *Enterococcus hirae* WEHI01, exopolysaccharide, immunomodulatory properties

## Abstract

Exopolysaccharide (EPS) from probiotic *Enterococcus hirae* WEHI01 was isolated and purified by anion exchange chromatography and gel chromatography, the results of which show that the EPS consists of four fractions, namely I01-1, I01-2, I01-3, and I01-4. As the main purification components, I01-2 and I01-4 were preliminarily characterized for their structure and their immunomodulatory activity was explored. The molecular weight of I01-2 was 2.28 × 10^4^ Da, which consists mainly of galactose, and a few other sugars including glucose, arabinose, mannose, xylose, fucose, and rhamnose, while the I01-4 was composed of galactose only and has a molecular weight of 2.59 × 10^4^ Da. Furthermore, the results of an evaluation of immunomodulatory activity revealed that I01-2 and I01-4 could improve the viability of macrophage cells, improve phagocytosis, boost NO generation, and encourage the release of cytokines including TNF-α and IL-6 in RAW 264.7 macrophages. These results imply that I01-2 and I01-4 could improve macrophage-mediated immune responses and might be useful in the production of functional food and medications.

## 1. Introduction

Exopolysaccharides (EPSs) are peculiar polymers of extracellular high-molecular-weight which can be produced by a variety of microorganisms (e.g., bacteria, fungi, and microalgae) [1]. EPSs isolated from lactic acid bacteria (LAB) are generally recognized as safe (GRAS) and are crucial natural additives in the food, cosmetic, and pharmaceutical industries [2,3]. EPS-producing LAB strains have great commercial potential due to their capability to enhance the rheology, texture, and mouthfeel of food [4,5,6,7]. Growing evidence demonstrated that EPSs from LAB possess various beneficial physiological effects including antioxidant [8], antimicrobial [9], antitumor [10], immunomodulatory [11], anti-biofilm [12], anti-viral [13], and cholesterol-lowering activities [14].

A growing number of publications demonstrated the immune-modulating effects of LAB-derived EPSs. Nikolic et al. reported that the EPS of *Lactobacillus paraplantarum* BGCG11 induced a significant immunoreaction [15]. The EPS of *Lactobacillus plantarum* JKL0142 could stimulate the immune activity of immunosuppressed mice macrophages [16]. Other reports demonstrated that LAB-derived EPSs increased particular cellular and humoral immune responses to antigens by promoting T/B-lymphocyte proliferation and boosting macrophage phagocytic activity, promoting the production of NO and cytokines [11,17,18]. In addition, the immunomodulatory activities of EPS are involved with their physicochemical properties such as their molecular weight and composition of monosaccharides [19].

Among EPS-producing LAB strains, in addition to the most studied *Lactobacillus* and *Bifidobacterium*, some *Enterococcus* strains are also known for their potential probiotic properties and desirable physical and chemical properties, and are preferred for use in many commercial probiotic feed additives to poultry and cattle [20,21]. Daillere et al. showed that the anti-tumor efficacy of cyclophosphamide relies on two gut commensal species, *Enterococcus hirae* and *Barnesiella intestinihominis* [22]. Hamid et al. found that adding *E. hirae* UPM02 to the diet of hybrid catfish successfully influenced immune responses and improved the expression of the immunity-related genes [23]. Recently, EPS from probiotic *Enterococcus* has also received more and more attention, on which most reports have focused on the EPS’s physicochemical characterization [24], antioxidant [25], antibiofilm [26] and anti-adhesion of the pathogen [27], but little is known about the immunomodulatory activity of EPS from probiotic enterococci. Our previous studies showed that the probiotic strain *E. hirae* WEHI01 isolated from healthy infants’ feces [28] was proven to alleviate inflammation, improve type 2 diabetes, regulate intestinal flora [29], and lower cholesterol [30] in rats. However, as an important component, the EPS of *E. hirae* WEHI01 has not yet been investigated, and its structure and function are still unknown.

In the host defense systems, macrophages are the bridge between innate and adaptive immunity [31]. Activated macrophages directly neutralize xenobiotics through phagocytosis and kill cancer cells and pathogenic microorganisms by secreting proinflammatory cytokines, including IL-1β, TNF-α, IL-6, and cytotoxic molecules NO [32,33]. Therefore, macrophages are considered to be crucial target cells in immunomodulatory effects.

In the present study, we purified EPS from *E. hirae* WEHI01 by using anion exchange chromatography and gel chromatography and characterized the primary structure by using Absolute Molecular Weight Analyzer, Fourier-transform infrared (FT-IR), and gas chromatography–mass spectrometry (GC-MS). Furthermore, we investigated the immunomodulatory activity of EPS fractions using a murine macrophage RAW 264.7 cell. This study aimed to reveal the primary structure of EPS from *E. hirae* WEHI01 and its capacity for immune regulation.

## 2. Materials and Methods

### 2.1. Materials and Reagents

The Cell Bank of the Chinese Academy of Sciences (Beijing, China) provided the murine macrophage cell line RAW 264.7 for use in research. Trimethylchlorosilane, hexamethyldisilane, pyridine, and trifluoroacetic acid (TFA) were obtained from Aladdin Biological Technology Co., Ltd. (Shanghai, China). Rhamnose, arabinose, xylose, fucose, mannose, glucose, and galactose were purchased from YuanYe Bio-Technology Co., Ltd. (Shanghai, China). Lipopolysaccharides (LPSs), Brain Heart Infusion (BHI), Mw cut-off 8000–14,000 Da MWCO membranes (MD34), and 0.1% neutral red stain solution were bought from Solarbio Life Science and Technology Co. Ltd. (Beijing, China). Bovine calf serum was purchased from Sigma Chemical Co., Ltd. (Saint Louis, MO, USA). ELISA kits for the analysis of TNF-α and IL-6 were purchased from Neobioscience Technology Co., Ltd. (Shenzhen, China). Cell Counting Kit-8 (CCK-8) was purchased from Beyotime Biotechnology Co. Ltd. (Shanghai, China). A kit for measuring nitric oxide (NO) was purchased from Nanjing Jiancheng Bioengineering Institute (Nanjing, China).

### 2.2. The Culture of Strain

The EPS-producing probiotic bacteria were previously isolated from healthy infant’s feces and named *E. hirae* WEHI01 [28], and cultured in BHI under anaerobic conditions at 37 °C.

### 2.3. Extraction, Production and Purification of EPS

Briefly, *E. hirae* WEHI01 was cultured in BHI for 20 h at 37 °C under anaerobic conditions, then underwent centrifugation at 9000× *g* for at least 5 min to collect supernatant and precipitated by mixing with two volumes of pre-cooled absolute ethyl alcohol. The precipitation was then collected by centrifugation (10,000× *g*, 20 min) and re-dissolved in Milli-Q water [27]. After deproteinized by Sevag reagent [34], dialysis of crude EPS against milli-Q water and lyophilized. To determine the yields of EPS, the supernatant (50 mL) was collected at different time intervals ranging from 0 to 50 h for EPS extraction. Using glucose as a standard, the EPS contents were determined by the phenol-sulfuric acid method and the absorbance was measured at 490 nm.

The purification of crude EPS according to our previous method, and the use of the phenol-sulfuric acid technique to determine the amount of carbohydrates in the eluate (2.0 mL/tube) [35]. Peak fractions were concentrated and further fractionated by a Superdex G-200 column (10 mm × 300 mm) and then eluted with 0.2 M NH_4_HCO_3_. The EPS fractions eluted in one peak were pooled together and dialyzed against ultrapure water, and finally concentrated by freeze-dried (SCIENTZ-10N, Ningbo SCIENTZ Biotechnology Co., LTD, China) for further analyses.

### 2.4. Structure Characterization of EPS

#### 2.4.1. Purity and Molecular Weight

The UV spectrum of I01-2 and I01-4 (0.1 mg/mL) was obtained on a U-3900 UV/VIS Spectrophotometer over a range of 200–600 nm. The homogeneity and molecular weight of EPS were determined using PL aquagel-OH MIXED (7.5 mm × 300 mm, 8 μm) (Aglient, Santa Rosa, CA, USA) equipped with Differential Refractometer (BI-DNDC/GPC, Brookhaven Inc., New York, NY, USA) and Molecular Weight Analyzer (BI-MwA, Brookhaven Inc., New York, NY, USA) according to our previous report [27].

#### 2.4.2. FT-IR Spectroscopy Analysis

FT-IR was recorded using the KBr-disks method [36] with the FT-IR spectrophotometer (Nicolet Nexus 470, Thermo Nicolet Co., Madison, WI, USA). The EPS measurement range was 400–4000 cm^−1^, as previously reported [37].

#### 2.4.3. Analysis of Monosaccharide Composition

With a slight modification based on our previously reported method [38], the monosaccharide composition was analyzed by GC-MS after acetylation. Firstly, I01-2, I01-4, and standard sugars were hydrolyzed with 2.0 M TFA at 110 °C for 4 h. The TFA residue was then removed by washing the hydrolysate twice with methanol and drying it under nitrogen. The product was then reduced with NaBH_4_, and acetylated with acetic anhydride (AC_2_O). To create the derivatives, chloroform was used to extract the substance. Then, the acetate derivatives were analyzed through GC-MS apparatus (Shimadzu GCMS-QP 2010, Japan) equipped with an RXI-5 SIL MS chromatographic column (30 m × 0.25 mm × 0.25 μm) (J&W Scientific, Folsom, CA, USA). The GC-MS operation was performed under the following conditions: Helium (carrier gas) at a constant flow velocity of 1.0 mL/min, injection temperature: 250 °C, initial column temperature: 120 °C and holding for 5 min, increasing to 250 °C at a rate of 3 °C/min, and holding at 250 °C for 5 min.

### 2.5. Immunocompetence Assays

#### 2.5.1. Cell Culture and Its Viability Assay

The murine macrophage cell line RAW 264.7 was cultured in DMEM supplemented with 10% bovine calf serum at 37 °C in an atmosphere of 5% CO_2_. Cells in 96-well plates (5.0 × 10^4^ cells/well) were treated with various concentrations (50, 100, 200, 400, 800. and 1000 μg/mL) of I01-2 and I01-4 for 24 h. Additionally, the proliferation of RAW 264.7 was detected by CCK-8 according to the manufacturer’s protocol. The absorbance was measured at 450 nm by a microplate reader (Varioskan Flash, Thermo Scientific, Waltham, MA, USA). Cell viability was calculated using the following equation:

$$\mathrm{Cell}\;\mathrm{viability}\;\left(\%\right)=\frac{A_{2}}{A_{1}}\times 100$$

where A_1_ is the absorbance of the blank group, and A_2_ is the absorbance of cells after treatment with EPS.

#### 2.5.2. Phagocytosis Assay and Morphology Observation

The phagocytic capacity of RAW 264.7 cells was evaluated by neutral red uptake assay [39]. The intervention of RAW 264.7 cells was made with various concentrations of I01-2, I01-4, and LPS (1 μg/mL) for 24 h in a 96-well plate. After washing the cells twice with Hanks, add 100 μL of 0.1% neutral red stain solution to each well. After incubation at 37 °C for another 3 h, the cells were washed three times with Hanks and then lysed by adding 200 μL lysate (anhydrous ethanol and acetic acid in a 1:1 ratio) to incubate for 1 h at room temperature. The absorbance at 540 nm was measured with a microplate reader. An optical microscope (Olympus, Tokyo, Japan) was used to observe the morphology.

#### 2.5.3. NO and Cytokines Secretion

Briefly, I01-2, I01-4, or LPS were incubated with RAW 264.7 cells in 6-well plates (1.0 × 10^6^ cells/well) for 12 h [40], and the quantities of NO, TNF-α and IL-6 in the supernatants were measured using commercial kits in accordance with the manufacturer’s instructions.

#### 2.5.4. Gene Expression Analysis by RT-qPCR

RAW 264.7 cells were handled as described above in 6-well plates. Following the manufacturer’s instructions, total RNA was obtained using the MiniBEST Universal RNA Extraction Kit. PrimeScript ^TM^ RT Reagent Kit with g DNA Eraser was used by the directions to create single-strand cDNA. Using SYBR Premix Ex Taq II kit quantitative real-time PCR (qPCR) was carried out to examine the transcription level of *iNOS*, *TNF-α,* and *IL-6* genes. The qPCR was run using the following cycling profile: preheating at 95 °C for 5 min, followed by 40 cycles of 95 °C for 30 s, 60 °C for 30 s, and 72 °C for 30 s. The 2^−ΔΔCt^ method was used to analyze real-time PCR, which was carried out in triplicate. The *β-actin* gene served as a reference gene. Table 1 contains a list of the primers used.

### 2.6. Statistical Analysis

At least three different replications of the experiment’s findings were made and all data were then presented as mean ± SD. Independent one-way ANOVA tests were utilized for statistical analysis in the Origin 2022 software (OriginLab, Northampton, MA, USA).

## 3. Results and Discussion

### 3.1. Extraction and Purification of EPS

**Figure 1:**
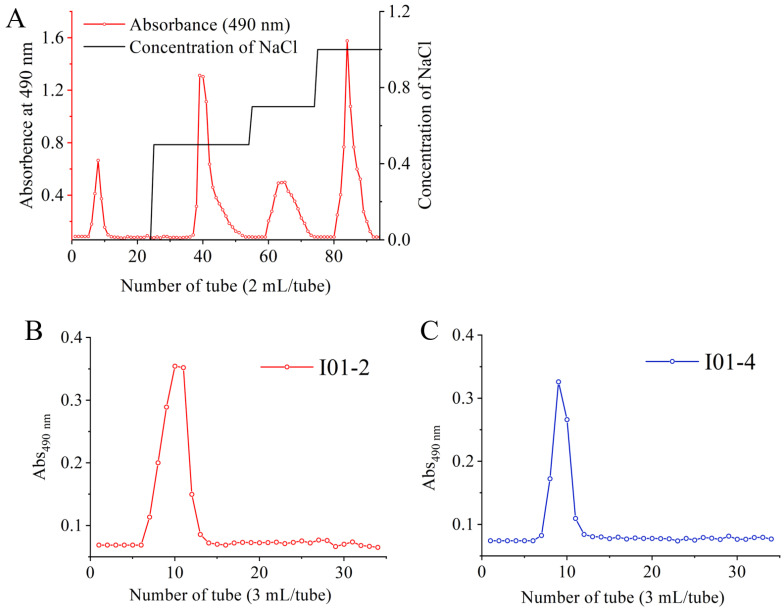
Elution profile of *E. hirae* WEHI01 crude EPS on HiTrap Q HP chromatography column with NaCl solutions (0, 0.5, 0.7, and 1 M) (**A**) and elution profile of I01-2 (**B**) and I01-4 (**C**) on Sephadex G-200 gel chromatography column with 0.2 M NH_4_HCO_3_ (**B**). –○–, 490 nm for the detection of carbohydrate.

EPS reached a maximum value of 606 mg/L, while the cell counts maintained 10.26 log CFU/mL at 30 h. The rough EPS was isolated from *E. hirae* WEHI01 and purified by chromatography. As shown in Figure 1A, four fractions (I01-1, I01-2, I01-3, and I01-4) were obtained by a HiTrap Q HP column and calculated by the number of crude EPS, the recovery rates of I01-1, I01-2, I01-3, and I01-4 were 12.2%, 21.6%, 8.1%, and 15.9%, respectively. The major fractions I01-2 and I01-4 were further purified by Sephadex G-200 gel permeation chromatography, and the result showed that each eluting peak was a separate fraction (Figure 1B,C), which was used for subsequent analysis. I01-1 is a neutral polysaccharide and the other three purified components are acidic polysaccharides. Similar results were observed in our previous study that the EPS from *E. faecium* WEFA23 was also composed of four fractions [27].

### 3.2. Mw and Monosaccharides Composition of I01-2 and I01-4

**Figure 2:**
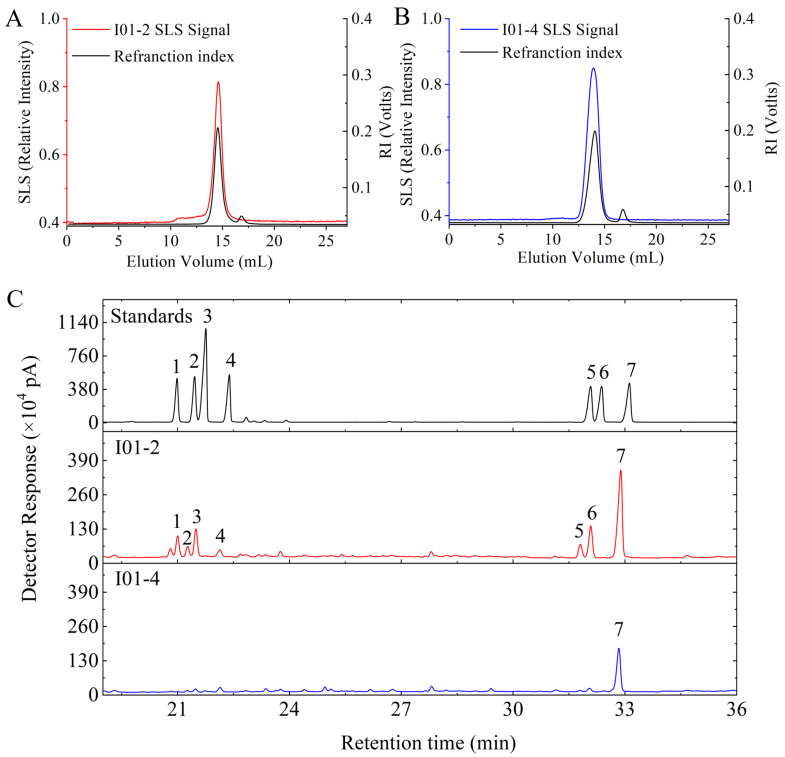
SEC-LLS analysis of I01-2 (**A**) and I01-4 (**B**) and GC chromatograms of the monosaccharide composition (**C**).

An Absolute Molecular Weight Analyzer was used to confirm the homogeneity and establish the average molecular weight distribution of I01-2 and I01-4. The curve of I01-2 and I01-4 were unimodal symmetrical peaks, as shown in Figure 2A,B. This indicates that the two fractions after purification were homogeneous, and the molecular weight of I01-2 and I01-4 was calculated to be 2.28 × 10^4^ and 2.59 × 10^4^ Da, respectively. By using GC-MS, the monosaccharide compositions of I01-2 and I01-4 were examined and their retention times were compared to reference sugar standards. As shown in Figure 2C, I01-2 was composed of galactose, glucose, arabinose, mannose, xylose, fucose, and rhamnose with a molar ratio of 1:0.296:0.262:0.120:0.08:0.08:0.048. However, the I01-4 fraction was composed of galactose only.

### 3.3. FT-IR Spectrum Analysis of I01-2 and I01-4

**Figure 3:**
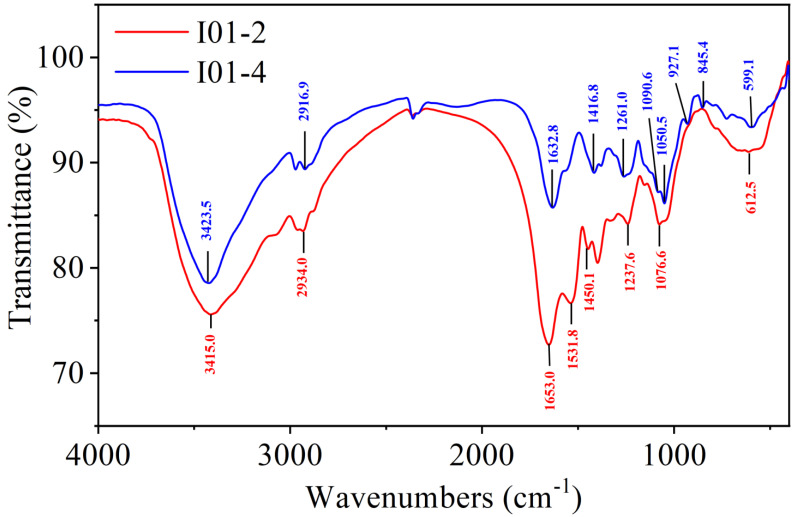
FT-IR spectra of I01-2 and I01-4 in the range of 400–4000 cm^−1^.

The FT-IR spectrum of I01-2 and I01-4 is shown in Figure 3. I01-2 and I01-4 exhibited intense and broad peaks around 3415 and 3423 cm^−1^, respectively, which were assigned to the O-H stretching vibrations’ absorption peaks of sugar compounds [41]. The peaks at 2934 and 2917 cm^−1^ were due to the C-H stretching vibration [42]. The carbonyl absorption peak is a strong C=O stretching absorption band in the 1900–1600 cm-1 region, and I01-2 and I01-4 each have an absorption peak at 1653 and 1632 cm^−1^, respectively, corresponding to the characteristic peak of C=O and indicating the presence of uronic acid [43]. Moreover, the absorption peaks at 1416 was attributed to the stretching vibrations of carboxylic groups (COO-), which indicated that the purified I01-4 was acidic polysaccharides, and the locations of these peaks was similar to the study of *Sibiraea laevigata (L.) Maxim* polysaccharides by FT-IR [44]. The 1260 cm^−1^ of I01-4 were assigned to O-H deformation vibrations [45]. The band between 1600 cm^−1^ and 1650 cm^−1^, which is assigned to the bending vibration of the coordinated water molecule, should be classified as a water band [46]. In addition, the absorption peaks at approximately 845 and 1090 cm^−1^ indicate the presence of the glycosidic bonds of both α and β configurations in I01-4 [47].

### 3.4. Immunomodulatory Activities of I01-2 and I01-4 on RAW264.7 Cells

#### 3.4.1. Effect of I01-2 and I01-4 on Cell Viability of RAW264.7

**Figure 4:**
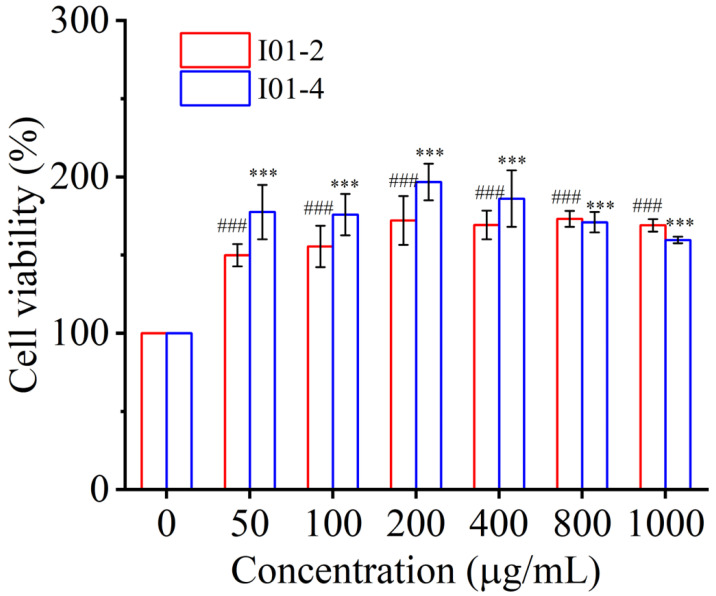
The effect of I01-2 and I01-4 on the viability of RAW 264.7 cells. Data were expressed as mean ± SEM. *** *p* < 0.001 (vs. I01-4 at concentrations of 0 μg/mL), ^###^ *p* < 0.001 (vs. I01-2at concentrations of 0 μg/mL).

Macrophages considered the crucial target cells for immunomodulatory effects, play a crucial role in the host’s first-line defense against various infections and cancer [48,49]. In the current study, the macrophage RAW 264.7 cell was used to evaluate the immunomodulatory activity of I01-2 and I01-4. By using the CCK-8 assay, the impact of I01-2 and I01-4 on cell viability was assessed. In Figure 4, I01-2 and I01-4 at concentrations of 50–1000 μg/mL exhibited non-toxicity to RAW 264.7 cells. On the other hand, both I01-2 and I01-4 increased the proliferation of RAW264.7 cells. The largest boosting effects for I01-2 and I01-4, respectively, were at 800 g/mL and 200 g/mL, reaching a maximum of 173.10% and 196.6%, respectively. Remarkably, the proliferation effect of I01-4 was obviously stronger than that of I01-2 at 50–400 μg/mL. Our result was in agreement with previous reports that EPS0142 (50–1000 μg/mL) from *L*. *plantarum* JLK0142 had no toxicity on RAW 264.7 cells [16] and EPS (5–1000 μg/mL) from *L*. *plantarum* NTU 102 promoted the cell viability of RAW 264.7 macrophages [50].

#### 3.4.2. Effects of I01-2 and I01-4 on Phagocytosis of RAW 264.7 Cells

**Figure 5:**
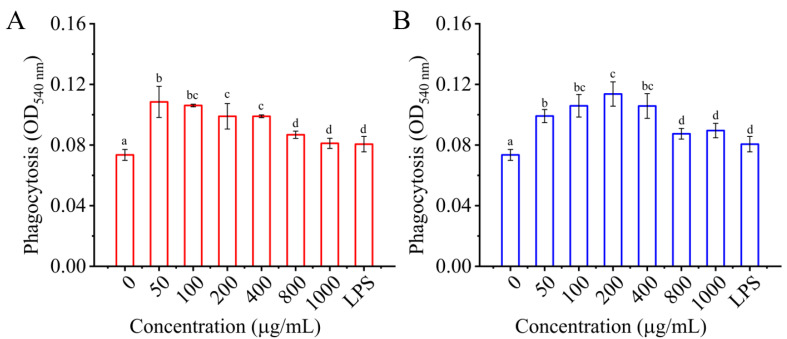
The effect of I01-2 (**A**) and I01-4 (**B**) on the phagocytosis of RAW 264.7 cells. The group was incubated with medium (0 μg/mL of I01-2 and I01-4) as a negative control and LPS (1.0 μg/mL) treatment as a positive control. Different superscript letters (a–d) indicate significant differences (*p* < 0.05) between the groups.

Phagocytosis is one of macrophage activation’s most distinguishing features [51]. Macrophages become antigen-presenting cells after phagocytic uptake and interplay with lymphocytes to modulate the adaptive immune response [52,53]. The effect of I01-2 and I01-4 on macrophage phagocytosis was measured by neutral red uptake assay in the present study. In contrast to the control, LPS dramatically increased the phagocytosis of RAW 264.7 cells, as seen in Figure 5. As for I01-2 and I01-4, the phagocytosis of RAW 264.7 cells was significantly higher than that of the negative control (0 μg/mL), with the strongest phagocytosis at a concentration of 50 μg/mL and 200 μg/mL, respectively. This result indicates that I01-2 and I01-4 enhanced the pinocytosis of RAW 264.7 cells, which was consistent with a previous report that EPS from *Lactobacillus* significantly improved the phagocytosis of RAW 264.7 cells [11,16,54].

#### 3.4.3. Effects of I01-2 and I01-4 on the RAW264.7 Cells’ Morphology

**Figure 6:**
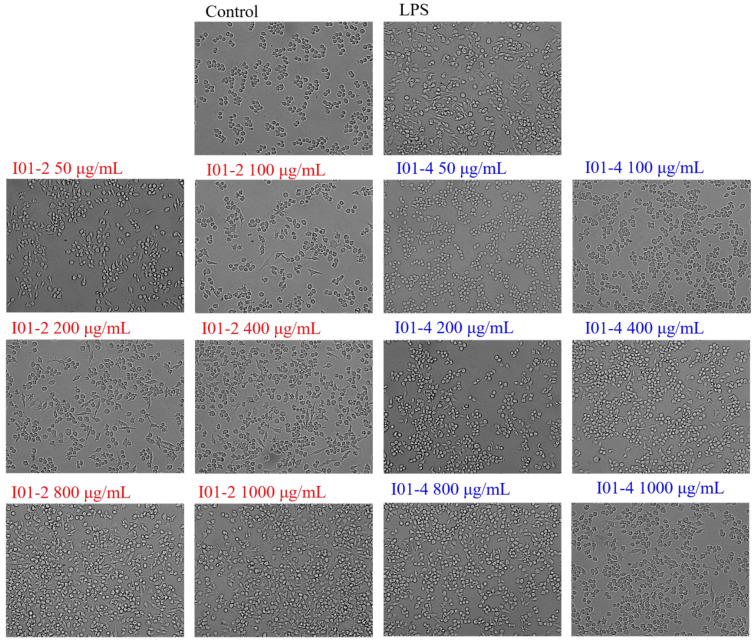
The effect of I01-2 and I01-4 on the morphology of RAW264.7 cells.

An inverted fluorescent microscope was employed to observe the RAW264.7 cells to determine whether I01-2 and I01-4 had any impact on their morphology. As shown in Figure 6, the RAW264.7 cells in the blank control group were round in shape and showed to be aggregated and growing under a white light 20× objective lens, whereas the cell morphology markedly changed to polygonal and dendritic when treated with LPS. Similarly, after treatment with I01-2 and I01-4, the morphology of RAW264.7 cells also showed concentration-dependent dendritic changes, and the morphological changes of RAW264.7 caused by I01-2 were more significant than those of I01-4.

#### 3.4.4. Effects of I01-2 and I01-4, Respectively, on the Generation of NO and the Secretion of IL-6 and TNF-α

**Figure 7:**
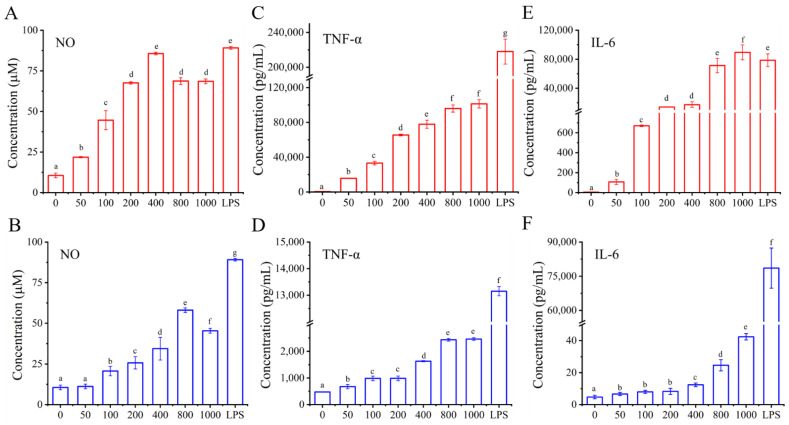
The effect of I01-2 and I01-4 on the production of NO (**A**,**B**), TNF-α (**C**,**D**), and IL-6 (**E**,**F**) in RAW 264.7 cells, respectively. The group incubated with medium only (0 μg/mL of I01-2 and I01-4) was used as a control. Different superscript letters (a–g) indicate significant differences (*p* < 0.05) between the groups.

Macrophages play a potential immunoregulatory role through the production of various mediators and cytokines and are therefore a significant part of host defense systems. While iNOS is a crucial NOS isoform that triggers NO synthesis, NO is an intracellular messenger molecule that plays a role in immunological responses and controls a diverse range of physiological processes, including the regulation of apoptosis [55,56,57,58]. Therefore, another indicator of macrophage activation in this investigation was the level of NO production. As can be seen from the data in Figure 7A,B, cells without EPS secreted a bit of NO, whereas I01-2 and I01-4 improved the production of NO at 50–400 μg/mL of I01-2 and 100–800 μg/mL of I01-4 in a dose-dependent manner, and reached the maximum of 85.67 and 59.12 μmol/L, respectively. Notably, however, excessive NO generation is hazardous and may cause apoptosis in macrophages [59]. As a result, we hypothesized that the decreased cell viability and phagocytic capacity of I01-2 and I01-4 with an increase in concentration from 400 μg/mL or 1000 μg/mL, respectively, might be caused by an excessive build-up of NO in macrophages. Additionally, the NO levels in groups receiving EPS treatments were weaker than those in the group receiving LPS treatments, indicating that the EPS effects were more moderate than that of LPS [60].

Activated macrophages can also produce a variety of cytokines other than NO that regulate cellular and humoral immune responses. TNF-α is a pleiotropic cytokine that regulates a wide spectrum of physiological processes, including cell proliferation, differentiation, apoptosis, and inflammation, and it is required for macrophage function [61,62]. However, IL-6 plays a key role in response signaling, which is associated with inflammatory regulation and antigen-presenting [63]. As shown in Figure 7C–F, the control group secreted a basal level of TNF-α and IL-6, while the intervention of I01-2 and I01-4 at all tested concentrations (50–1000 μg/mL) resulted in a remarkable (*p* < 0.05) increase in a dose-dependent manner, in which I01-2 showed better immune activity than I01-4—and its IL-6 and TNF-α contents reached maximums of 8.64 × 10^4^ and 1.01 × 10^5^ pg/mL, respectively, which were 1977 and 41 times those of I01-4, respectively. Similar results have been found that *L*. *plantarum* NTU 102-EPS exhibited strong immunomodulatory activities at the level of TNF-α and IL-6 [50] as EPS from *L*. *helveticus* LZ-R-5 enhanced the immunological activity by stimulating the secretion of TNF-α, IL-1β, and IL-6 in RAW264.7 [11]. However, EPS from *L. rhamnosus* KL37 could induce the release of IL-10 in RAW 264.7 cells [64].

**Figure 8:**
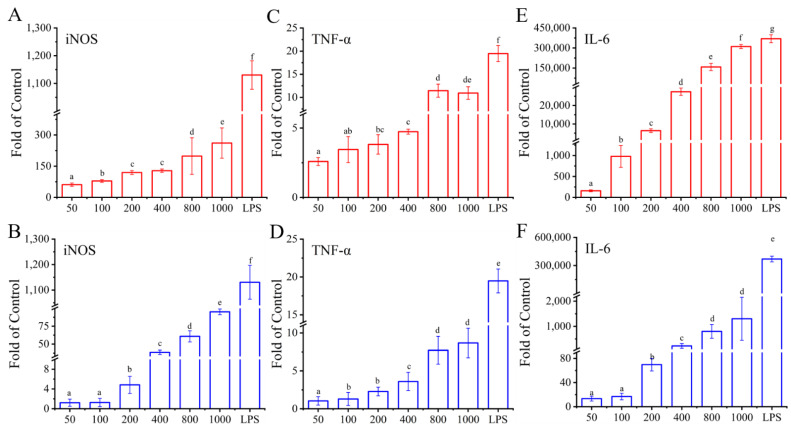
The effects of I01-2 and I01-4 on mRNA levels of *iNOS* (**A**,**B**), *TNF-α* (**C**,**D**), *IL-6* (**E**,**F**) in RAW 264.7 cells, respectively. Different superscript letters (a–g) indicate significant differences (*p* < 0.05) between the groups.

Previous studies have confirmed that the activation of macrophages is regulated by immune-related genes [65]. To confirm the effects of I01-2 and I01-4 on the mRNA expression of cytokines in this study, RT-qPCR was used to detect the gene transcription level of *iNOS*, *TNF-α*, and *IL-6*. In Figure 8A–F, the mRNA levels of *iNOS*, *TNF-α* and *IL-6* also showed a significant increase in cells treated with I01-2, I01-4, or LPS when compared to the control group, which was consistent with the NO, TNF-α, and IL-6 secretion levels. It was also found that the EPS from *L. plantarum* RS20D could up-regulate pro-inflammatory cytokines at the mRNA level [66]. Furthermore, all I01-2 and I01-4 treated groups had lower levels of *iNOS*, *TNF-α,* and *IL-6* at the mRNA level than that of the LPS-treated group (*p* < 0.05), which is consistent with the results corresponding to NO production, TNF-α, and IL-6 secretion. Studies demonstrated a relationship between the structural traits of polysaccharides and their biological activity, including the chemical make-up, molecular weight, conformation, glycosidic linkages, and degree of branching [39]. The structure of EPS, in terms of functional groups and glycosidic bonds, is very intimately related to their immunomodulatory activities [26]. A high level of immunomodulatory action was noted in some acidic, galactose-rich EPS, according to several publications [57,67,68]. Hidalgo-Cantabrana et al. reported that EPSs with a negative charge and/or small size can operate as mild stimulators of immune cells and that the galactose content of EPSs may enhance their immunomodulatory effects on the macrophages [69,70]. In our study, I01-2 exhibited an immunomodulatory activity superior to that of I01-4, which may also be due to their differences in monosaccharide composition. All of these findings suggested that I01-2 and I01-4 could cause macrophages to produce more NO, TNF-α, and IL-6, therefore improving the immunological activity, which might play a protective role in host defense against infections or cancer.

## 4. Conclusions

In the present study, the production, purification, characterization, and immunomodulatory activity of EPS from *E. hirae* WEHI01 were investigated in vitro. I01-2 and I01-4, which were major fractions therein, were described for their preliminary structure and in vitro immunomodulatory activities. I01-2 and I01-4 are acidic polysaccharides with molecular weights of 2.28 × 10^4^ and 2.59 × 10^4^ Da, respectively. The composition of I01-2 was mainly composed of galactose and a few other sugars, namely galactose, glucose, arabinose, mannose, xylose, fucose, and rhamnose, while galactose only constituted I01-4. Additionally, I01-2 and I01-4 also showed strong immunomodulatory action by accelerating macrophage phagocytosis, producing more NO, and encouraging the release of TNF-α and IL-6 in RAW 264.7 cells. According to all of these findings, I01-2 and I01-4 demonstrated immunomodulatory action and might have positive effects on the production of functional foods and medications.

## Figures and Tables

**Table 1:** Primers used for qPCR.

Genes	Forward Primer (5′-3′)	Reverse Primer (5′-3′)
*iNOS*	GCGAAAGGTCATGGCTTCAC	TCTTCCAAGGTGCTTGCCTT
*TNF-α*	CGAGTGACAAGCCTGTAGCC	ACAAGGTACAACCCATCGGC
*IL-6*	GTCCTTCCTACCCCAATTTCCA	CGCACTAGGTTTGCCGAGTA
*β-actin*	GCTCCTCCTGAGCGCAAGTA	CAGCTCAGTAACAGTCCGCC

## Data Availability

The data used to support the findings of this study can be made available by the corresponding author upon request.

## References

[1] Freitas F., Torres C.A., Reis M.A. (2017). Engineering aspects of microbial exopolysaccharide production. Bioresour. Technol..

[2] Lynch K.M., Coffey A., Arendt E.K. (2018). Exopolysaccharide producing lactic acid bacteria: Their techno-functional role and potential application in gluten-free bread products. Food Res. Int..

[3] Buksa K., Kowalczyk M., Boreczek J. (2021). Extraction, purification and characterisation of exopolysaccharides produced by newly isolated lactic acid bacteria strains and the examination of their influence on resistant starch formation. Food Chem..

[4] Lynch K.M., McSweeney P.L., Arendt E.K., Uniacke-Lowe T., Galle S., Coffey A. (2014). Isolation and characterisation of exopolysaccharide-producing Weissella and Lactobacillus and their application as adjunct cultures in Cheddar cheese. Int. Dairy J..

[5] Tiwari S., Kavitake D., Devi P.B., Shetty P.H. (2021). Bacterial exopolysaccharides for improvement of technological, functional and rheological properties of yoghurt. Int. J. Biol. Macromol..

[6] Bachtarzi N., Kharroub K., Ruas-Madiedo P. (2019). Exopolysaccharide-producing lactic acid bacteria isolated from traditional Algerian dairy products and their application for skim-milk fermentations. LWT.

[7] Saadat Y.R., Khosroushahi A.Y., Gargari B.P. (2019). A comprehensive review of anticancer, immunomodulatory and health beneficial effects of the lactic acid bacteria exopolysaccharides. Carbohydr. Polym..

[8] Zhang Z., Liu Z., Tao X., Wei H. (2016). Characterization and sulfated modification of an exopolysaccharide from Lactobacillus plantarum ZDY2013 and its biological activities. Carbohydr. Polym..

[9] Ayyash M., Abu-Jdayil B., Itsaranuwat P., Galiwango E., Tamiello-Rosa C., Abdullah H., Esposito G., Hunashal Y., Obaid R.S., Hamed F. (2019). Characterization, bioactivities, and rheological properties of exopolysaccharide produced by novel probiotic Lactobacillus plantarum C70 isolated from camel milk. Int. J. Biol. Macromol..

[10] Jiang B., Tian L., Huang X., Liu Z., Jia K., Wei H., Tao X. (2020). Characterization and antitumor activity of novel exopolysaccharide APS of Lactobacillus plantarum WLPL09 from human breast milk. Int. J. Biol. Macromol..

[11] You X., Li Z., Ma K., Zhang C., Chen X., Wang G., Yang L., Dong M., Rui X., Zhang Q. (2020). Structural characterization and immunomodulatory activity of an exopolysaccharide produced by Lactobacillus helveticus LZ-R-5. Carbohydr. Polym..

[12] Sarikaya H., Aslim B., Yuksekdag Z. (2016). Assessment of anti-biofilm activity and bifidogenic growth stimulator (BGS) effect of lyophilized exopolysaccharides (l-EPSs) from *Lactobacilli* strains. Int. J. Food Prop..

[13] Biliavska L., Pankivska Y., Povnitsa O., Zagorodnya S. (2019). Antiviral Activity of Exopolysaccharides Produced by Lactic Acid Bacteria of the Genera Pediococcus, Leuconostoc and Lactobacillus against Human Adenovirus Type 5. Medicina.

[14] Yılmaz T., Şimşek Ö. (2020). Potential Health Benefits of Ropy Exopolysaccharides Produced by *Lactobacillus plantarum*. Molecules.

[15] Nikolic M., López P., Strahinic I., Suárez A., Kojic M., Fernández-García M., Topisirovic L., Golic N., Ruas-Madiedo P. (2012). Characterisation of the exopolysaccharide (EPS)-producing Lactobacillus paraplantarum BGCG11 and its non-EPS producing derivative strains as potential probiotics. Int. J. Food Microbiol..

[16] Wang J., Wu T., Fang X., Min W., Yang Z. (2018). Characterization and immunomodulatory activity of an exopolysaccharide produced by Lactobacillus plantarum JLK0142 isolated from fermented dairy tofu. Int. J. Biol. Macromol..

[17] Khalil M.A., Sonbol F.I., Al-Madboly L.A., Aboshady T.A., Alqurashi A.S., Ali S.S. (2022). Exploring the therapeutic potentials of exopolysaccharides derived from lactic acid bacteria and bifidobacteria: Antioxidant, antitumor, and periodontal regeneration. Front. Microbiol..

[18] Park H.-R., Hwang D., Suh H.-J., Yu K.-W., Kim T.Y., Shin K.-S. (2017). Antitumor and antimetastatic activities of rhamnogalacturonan-II-type polysaccharide isolated from mature leaves of green tea via activation of macrophages and natural killer cells. Int. J. Biol. Macromol..

[19] Hidalgo-Cantabrana C., Nikolic M., López P., Suárez A., Miljkovic M., Kojic M., Margolles A., Golic N., Ruas-Madiedo P. (2014). Exopolysaccharide-producing Bifidobacterium animalis subsp. lactis strains and their polymers elicit different responses on immune cells from blood and gut associated lymphoid tissue. Anaerobe.

[20] Lodemann U., Hübener K., Jansen N., Martens H. (2006). Effects of *Enterococcus faecium* NCIMB 10415 as probiotic supplement on intestinal transport and barrier function of piglets. Arch. Anim. Nutr..

[21] Awad W., Ghareeb K., Böhm J. (2008). Intestinal Structure and Function of Broiler Chickens on Diets Supplemented with a Synbiotic Containing Enterococcus faecium and Oligosaccharides. Int. J. Mol. Sci..

[22] Daillère R., Vétizou M., Waldschmitt N., Yamazaki T., Isnard C., Poirier-Colame V., Duong C.P.M., Flament C., Lepage P., Roberti M.P. (2016). Enterococcus hirae and Barnesiella intestinihominis Facilitate Cyclophosphamide-Induced Therapeutic Immunomodulatory Effects. Immunity.

[23] Hamid N.H., Daud H.M., Kayansamruaj P., Abu Hassim H., Yusoff S.M., Abu Bakar S.N., Srisapoome P. (2021). Short- and long-term probiotic effects of Enterococcus hirae isolated from fermented vegetable wastes on the growth, immune responses, and disease resistance of hybrid catfish (Clarias gariepinus × Clarias macrocephalus). Fish Shellfish Immunol..

[24] Jayamanohar J., Devi P.B., Kavitake D., Rajendran S., Priyadarisini V.B., Shetty P.H. (2018). Characterization of α-D-glucan produced by a probiont Enterococcus hirae KX577639 from feces of south Indian Irula tribals. Int. J. Biol. Macromol..

[25] Bhat B., Bajaj B.K. (2018). Hypocholesterolemic and bioactive potential of exopolysaccharide from a probiotic Enterococcus faecium K1 isolated from kalarei. Bioresour. Technol..

[26] Ferreira S.S., Passos C.P., Madureira P., Vilanova M., Coimbra M.A. (2015). Structure–function relationships of immunostimulatory polysaccharides: A review. Carbohydr. Polym..

[27] Jia K., Tao X., Liu Z., Zhan H., He W., Zhang Z., Zeng Z., Wei H. (2018). Characterization of novel exopolysaccharide of Enterococcus faecium WEFA23 from infant and demonstration of its in vitro biological properties. Int. J. Biol. Macromol..

[28] Zhang F., Jiang M., Wan C., Chen X., Chen X., Tao X., Shah N.P., Wei H. (2016). Screening probiotic strains for safety: Evaluation of virulence and antimicrobial susceptibility of enterococci from healthy Chinese infants. J. Dairy Sci..

[29] Wei M., Gu E., Luo J., Zhang Z., Xu D., Tao X., Shah N.P., Wei H. (2020). Enterococcus hirae WEHI01 isolated from a healthy Chinese infant ameliorates the symptoms of type 2 diabetes by elevating the abundance of Lactobacillales in rats. J. Dairy Sci..

[30] Zhang F., Qiu L., Xu X., Liu Z., Zhan H., Tao X., Shah N.P., Wei H. (2017). Beneficial effects of probiotic cholesterol-lowering strain of Enterococcus faecium WEFA23 from infants on diet-induced metabolic syndrome in rats. J. Dairy Sci..

[31] Panda S., Ding J.L. (2014). Natural Antibodies Bridge Innate and Adaptive Immunity. J. Immunol..

[32] Zhang M., Yan M., Yang J., Li F., Wang Y., Feng K., Wang S., Lin N., Wang Y., Yang B. (2022). Structural characterization of a polysaccharide from Trametes sanguinea Lloyd with immune-enhancing activity via activation of TLR4. Int. J. Biol. Macromol..

[33] Cheng X.-D., Wu Q.-X., Zhao J., Su T., Lu Y.-M., Zhang W.-N., Wang Y., Chen Y. (2019). Immunomodulatory effect of a polysaccharide fraction on RAW 264.7 macrophages extracted from the wild Lactarius deliciosus. Int. J. Biol. Macromol..

[34] Zhang H.-L., Cui S.-H., Zha X.-Q., Bansal V., Xue L., Li X.-L., Hao R., Pan L.-H., Luo J.-P. (2014). Jellyfish skin polysaccharides: Extraction and inhibitory activity on macrophage-derived foam cell formation. Carbohydr. Polym..

[35] Xie Y., Zhou R.-R., Xie H.-L., Yu Y., Zhang S.-H., Zhao C.-X., Huang J.-H., Huang L.-Q. (2018). Application of near infrared spectroscopy for rapid determination the geographical regions and polysaccharides contents of Lentinula edodes. Int. J. Biol. Macromol..

[36] Li C., Fu X., Luo F., Huang Q. (2012). Effects of maltose on stability and rheological properties of orange oil-in-water emulsion formed by OSA modified starch. Food Hydrocoll..

[37] Liu C., Zhang D., Shen Y., Tao X., Liu L., Zhong Y., Fang S. (2015). DPF2 regulates OCT4 protein level and nuclear distribution. Biochim. Biophys. Acta.

[38] Liu J., Zhang C., Wang Y., Yu H., Liu H., Wang L., Yang X., Liu Z., Wen X., Sun Y. (2011). Structural elucidation of a heteroglycan from the fruiting bodies of Agaricus blazei Murill. Int. J. Biol. Macromol..

[39] Yu Y., Shen M., Wang Z., Wang Y., Xie M., Xie J. (2017). Sulfated polysaccharide from Cyclocarya paliurus enhances the immunomodulatory activity of macrophages. Carbohydr. Polym..

[40] Zhang M., Wang G., Lai F., Wu H. (2016). Structural Characterization and Immunomodulatory Activity of a Novel Polysaccharide from *Lepidium meyenii*. J. Agric. Food Chem..

[41] Wang Y., Li C., Liu P., Ahmed Z., Xiao P., Bai X. (2010). Physical characterization of exopolysaccharide produced by Lactobacillus plantarum KF5 isolated from Tibet Kefir. Carbohydr. Polym..

[42] Wang X., Shao C., Liu L., Guo X., Xu Y., Lü X. (2017). Optimization, partial characterization and antioxidant activity of an exopolysaccharide from Lactobacillus plantarum KX041. Int. J. Biol. Macromol..

[43] Isfahani F.M., Tahmourespour A., Hoodaji M., Ataabadi M., Mohammadi A. (2018). Characterizing the new bacterial isolates of high yielding exopolysaccharides under hypersaline conditions. J. Clean. Prod..

[44] Yang X.H., Yang J.T., Liu H.H., Ma Z.R., Guo P.H., Chen H., Gao D.D. (2022). Extraction, structure analysis and antioxidant activity of Sibiraea laevigata (L.) Maxim polysaccharide. Int. J. Food Prop..

[45] Wang Y., Mao F., Wei X. (2012). Characterization and antioxidant activities of polysaccharides from leaves, flowers and seeds of green tea. Carbohydr. Polym..

[46] Liu J., Luo J., Ye H., Sun Y., Lu Z., Zeng X. (2009). Production, characterization and antioxidant activities in vitro of exopolysaccharides from endophytic bacterium Paenibacillus polymyxa EJS-3. Carbohydr. Polym..

[47] Kozarski M., Klaus A., Niksic M., Jakovljevic D., Helsper J.P., Van Griensven L.J. (2011). Antioxidative and immunomodulating activities of polysaccharide extracts of the medicinal mushrooms Agaricus bisporus, Agaricus brasiliensis, Ganoderma lucidum and Phellinus linteus. Food Chem..

[48] Wang G., Zhu L., Yu B., Chen K., Liu B., Liu J., Qin G., Liu C., Liu H., Chen K. (2016). Exopolysaccharide from Trichoderma pseudokoningii induces macrophage activation. Carbohydr. Polym..

[49] Wang S., Liu R., Yu Q., Dong L., Bi Y., Liu G. (2019). Metabolic reprogramming of macrophages during infections and cancer. Cancer Lett..

[50] Liu C.-F., Tseng K.-C., Chiang S.-S., Lee B.-H., Hsu W.-H., Pan T.-M. (2011). Immunomodulatory and antioxidant potential of Lactobacillus exopolysaccharides. J. Sci. Food Agric..

[51] Li C., Li X., You L., Fu X., Liu R.H. (2017). Fractionation, preliminary structural characterization and bioactivities of polysaccharides from Sargassum pallidum. Carbohydr. Polym..

[52] Feng L., Yin J., Nie S., Wan Y., Xie M. (2016). Fractionation, physicochemical property and immunological activity of polysaccharides from Cassia obtusifolia. Int. J. Biol. Macromol..

[53] Schepetkin I.A., Quinn M.T. (2006). Botanical polysaccharides: Macrophage immunomodulation and therapeutic potential. Int. Immunopharmacol..

[54] Xu Y., Cui Y., Wang X., Yue F., Shan Y., Liu B., Zhou Y., Yi Y., Lü X. (2019). Purification, characterization and bioactivity of exopolysaccharides produced by Lactobacillus plantarum KX041. Int. J. Biol. Macromol..

[55] Wen Z.-S., Xiang X.-W., Jin H.-X., Guo X.-Y., Liu L.-J., Huang Y.-N., OuYang X.-K., Qu Y.-L. (2016). Composition and anti-inflammatory effect of polysaccharides from Sargassum horneri in RAW264.7 macrophages. Int. J. Biol. Macromol..

[56] Alderton W.K., Cooper C.E., Knowles R.G. (2001). Nitric oxide synthases: Structure, function and inhibition. Biochem. J..

[57] Wang W., Zou Y., Li Q., Mao R., Shao X., Jin D., Zheng D., Zhao T., Zhu H., Zhang L. (2016). Immunomodulatory effects of a polysaccharide purified from Lepidium meyenii Walp. on macrophages. Process Biochem..

[58] Wang M., Zhu P., Zhao S., Nie C., Wang N., Du X., Zhou Y. (2017). Characterization, antioxidant activity and immunomodulatory activity of polysaccharides from the swollen culms of Zizania latifolia. Int. J. Biol. Macromol..

[59] Nie C., Zhu P., Ma S., Wang M., Hu Y. (2018). Purification, characterization and immunomodulatory activity of polysaccharides from stem lettuce. Carbohydr. Polym..

[60] Zheng D., Zou Y., Cobbina S.J., Wang W., Li Q., Chen Y., Feng W., Zou Y., Zhao T., Zhang M. (2016). Purification, characterization and immunoregulatory activity of a polysaccharide isolated from *Hibiscus sabdariffa* L.. J. Sci. Food Agric..

[61] Habijanic J., Berovic M., Boh B., Plankl M., Wraber B. (2015). Submerged cultivation of Ganoderma lucidum and the effects of its polysaccharides on the production of human cytokines TNF-α, IL-12, IFN-γ, IL-2, IL-4, IL-10 and IL-17. New Biotechnol..

[62] Aggarwal B.B. (2003). Signalling pathways of the TNF superfamily: A double-edged sword. Nat. Rev. Immunol..

[63] Mihara M., Hashizume M., Yoshida H., Suzuki M., Shiina M. (2011). IL-6/IL-6 receptor system and its role in physiological and pathological conditions. Clin. Sci..

[64] Ciszek-Lenda M., Nowak B., Śróttek M., Gamian A., Marcinkiewicz J. (2011). Immunoregulatory potential of exopolysaccharide from Lactobacillus rhamnosus KL37. Effects on the production of inflammatory mediators by mouse macrophages. Int. J. Exp. Pathol..

[65] Wang L., Nie Z.-K., Zhou Q., Zhang J.-L., Yin J.-J., Xu W., Qiu Y., Ming Y.-L., Liang S. (2014). Antitumor efficacy in H22 tumor bearing mice and immunoregulatory activity on RAW 264.7 macrophages of polysaccharides from Talinum triangulare. Food Funct..

[66] Zhu Y., Wang X., Pan W., Shen X., He Y., Yin H., Zhou K., Zou L., Chen S., Liu S. (2018). Exopolysaccharides produced by yogurt-texture improving Lactobacillus plantarum RS20D and the immunoregulatory activity. Int. J. Biol. Macromol..

[67] Georgiev Y.N., Ognyanov M.H., Kiyohara H., Batsalova T.G., Dzhambazov B.M., Ciz M., Denev P.N., Yamada H., Paulsen B.S., Vasicek O. (2017). Acidic polysaccharide complexes from purslane, silver linden and lavender stimulate Peyer’s patch immune cells through innate and adaptive mechanisms. Int. J. Biol. Macromol..

[68] Wang M., Zhao S., Zhu P., Nie C., Ma S., Wang N., Du X., Zhou Y. (2018). Purification, characterization and immunomodulatory activity of water extractable polysaccharides from the swollen culms of Zizania latifolia. Int. J. Biol. Macromol..

[69] Hidalgo-Cantabrana C., Lopez-Suarez P., Gueimonde M., Reyes-Gavilan C.D.L., Suarez-Diaz A.M., Margolles A., Ruas-Madiedo P. (2012). Immune Modulation Capability of Exopolysaccharides Synthesised by Lactic Acid Bacteria and Bifidobacteria. Probiotics Antimicrob. Proteins.

[70] Chen Y.-C., Wu Y.-J., Hu C.-Y. (2019). Monosaccharide composition influence and immunomodulatory effects of probiotic exopolysaccharides. Int. J. Biol. Macromol..

